# Training Public Health Professionals to Tackle Women, Children, and Families Health Issues: A “Skill-Based Qualitative Learning Approach”

**DOI:** 10.3389/fpubh.2021.743176

**Published:** 2021-09-16

**Authors:** Cecilia Obeng

**Affiliations:** Department of Applied Health Science, School of Public Heath, Indiana University, Bloomington, IN, United States

**Keywords:** qualitative, phenomenology, skill-based learning, teaching approach, workforce training

## Abstract

**Purpose:** There are several teaching and learning approaches but finding the one that is appropriate for a particular field or training program is an arduous task. The purpose of this paper is to introduce the “*Skill Based Qualitative Learning Approach*” (SBQLA) in training health professionals.

**Description:** The SBQLA is a pedagogical approach *via* which learners are trained in developing qualitative questionnaires and interview skills to learn from experts in the Public Health (PH) field. This teaching approach arms students with interview skills that help them identify and address PH roadblocks and get them authentic information from experts. It also equips them with techniques on how to do formalized presentations and come up with projects and interventions that help mitigate and eliminate drivers of health problems among women, children and families.

**Assessment:** Learners' field experiences are shared in a professional presentation style in a class to help trainees benefit from each other's information and to get formalized feedback on their presentation. Assessment in this learning approach is based on a synthesis and an analysis of data collected from professionals.

**Conclusion:** Findings from this learning approach enables experts to shed light on true stories shared by real and authentic individuals whose faces can be associated with their shared experiences. This learning approach makes it possible for trainees to also initiate projects that help them tackle existing and emerging public health issues in their future work.

## Introduction

Teaching/evaluation method selection depends on the subject matter being communicated and the method's effectiveness on students' learning. The appropriateness of a specific teaching method is considered in connection with the needs of the learners and what they will achieve (1).

The commonly used teaching approach, the *didactic method*, is a teacher-centered approach where all aspects of the teaching/learning information are selected by the teacher (2). Finding the alternative teaching/learning approach for preparing a particular cohort of workforce should be done in correlation with the workforce needs of trainees. Any such training must attract, hold, and build on the trainees' interest in a particular identified area and this is where the pedagogical methodology of qualitative orientation comes in. My own experience spanning nearly two decades in university teaching indicated that the “*Skill Based Qualitative Learning Approach*” (SBQLA) helps students search for and select (*via* interviewing experts in the field) areas in PH that they are interested in, and will be comfortable working in, during their daily professional routines. It is common knowledge that one cannot help others well if one does not have interest in the chosen area. A trainers' interest and expertise in qualitative method of learning is therefore central to employing it as a pedagogical or analytical training tool and/or approach.

### What Is Skill-Based Qualitative Learning Approach?

The SBQLA in Public Health (PH) trains PH workforce trainees in developing qualitative questions and interview skills for soliciting and/or eliciting information from PH leaders. Trainees rigorously interview experts about their leadership strategies and learn about what works for them. This type of qualitative interview approach explores the accomplishments that leaders have achieved in doing their work, the problems they encountered, and the coping mechanisms they put in place to ensure some measure of success, among others.

In this learning approach, trainees develop qualitative questions with help from qualitative research experts to help guide their interviews. These questions/questionnaires are validated before their use. Meticulous interview skills are also taught to the trainees.

## Method, Approach, and Results

This teaching method/idea is qualitative in nature and was started in 2011 when the author developed a course titled, “Introduction to Qualitative Inquiry in Public Health Research,” in her School of Public Health. The goal of this teaching approach was to ensure that students will be able to synthesize and analyze qualitative research data and critically analyze major concepts, themes, or issues associated with the qualitative research data. In pursuing this objective, students were introduced to the major issues and academic writings on qualitative research. Important among these were research design, data collection and analysis. Student trainees were also taken through the various qualitative research theories and methods.

Thus far, 132 trainees have taken courses in which the SBQLA method was used. Some participants' selected topics are provided in Table 1. More specific details are presented in the results section of this paper.

**Table 1:** Successful topics selected by students.

Women's issues	❖ Maternal mortality in the US ❖ Prenatal care issues and breastfeeding ❖ Anxiety and depression among new mothers ❖ Breastfeeding and women with health problems ❖ Public health education and breastfeeding in public ❖ Coronavirus pandemic, women, and children's health issues.
Children and youth issues	❖ Infant mortality in the US ❖ Sexual health education among youth ❖ Teenage pregnancy and child health
Family issues	❖ Insurance and women and child health issues ❖ Child injuries, abuse, neglect and maltreatment ❖ Childbirth and support for families

### Why Is Using Qualitative Interview Approach Important in Training PH Workforce to Tackle PH Issues

Qualitative research elicits narratives from study participants with the view to understanding their experiences, views, beliefs, thoughts, opinions etc. It is also used in generating ideas for an investigation or inquire into a problem (3–5).

Currently, qualitative research approach is being used in the training of our Maternal and Child Health (MCH) trainees to help them to understand the perspectives of experts in the field, and hence, learn from the studied participants. Such an approach also enables our trainees to contextualize issues affecting the studied participants' unique social, cultural and political ecologies and sheds light on how they interrogate, change or transform the social conditions within which they operate [see, (6)].

The MCH trainees are trained to develop and use open-ended questions in their interviews. Once the data are gathered, they are assisted in categorizing their collected data into themes. Thematizing the responses helps the trainees to make sense of their studied participants' perspectives (3, 4).

This qualitative approach of learning allows trainees to get ideas that have authentic sources and are original, given the fact that such ideas emerge from the interviewed professionals experts (3–5, 7).

The authenticity and credence of the gathered ideas also help the trainees in their job journey given their originality, tested-and-proven quality, and applicability to real life situations in the lives of MCH professionals.

Furthermore, this kind of first-hand learning approach gives trainees the chance to craft their questions for their future job needs and to get answers from experts from whom they learn. The use of this approach also helps learners to get firsthand information about the nature of the job, anticipated challenges, and ways of dealing with such challenges in their future jobs.

### Reason for Using Phenomenology in MCH Workforce Training

The qualitative method used in training our MCH workforce was crafted within the phenomenological approach. Working within phenomenological methodology required us to gather data through interviewing our studied participants (5, 7) who had experienced a phenomenon of interest to the interviewers. Such studied participants were selected and interviewed with the view to soliciting important firsthand information about their everyday “lived experiences,” things they anticipated as well as those that were unexpected. Other important information sought included problems the studied participants faced, how they dealt with coworkers, job difficulties or obstacles and how these overall experiences helped to shape their working lives.

Also, of importance is the fact that in phenomenological approach to training, learners are trained to place emphasis on study participants' experiential narratives and the meanings such participants attribute to their narratives rather than the trainees' own explanations of such experiences (3, 5, 7). In MCH, this kind of approach to learning helps trainees to learn from MCH experts' lived experiences, what they have gone through and how they dealt with specific and unique issues that arose in such encounters. Gaining such experience *via* the studied participants contributes to trainees' future work knowledge. In particular, it prepares them for the possible occurrence of some of the issues their MCH studied participants encountered and how they coped with them thereby equipping them (the trainees) with the acquired skill to deal with or handle any such issues should they arise in the future.

*A typical strategy used in a phenomenological study involves:*

A) Using semi-structured open-ended non-leading questions that help participants describe their “lived experiences” and probe them for detailed experiences when necessary.
B) Doing the interviews until saturation is reached. Saturation in qualitative research is when new interviewees are giving already given answers.
C) Having at least two people independently coding the data and meeting to agree on common themes.
D) Checking validity and reliability by going back to interviewees to confirm information; and
E) Categorizing and describing the MCH professionals lived experiences in themes to enable the trainees to identify the “essence” of their lived experiences (3, 7).

### How Is This Approach Introduced in an MCH Trainee's Curriculum?

From my own experience teaching qualitative research in public health and teaching the MCH Foundational course using SBQLA, the following curricular strategies have been identified as pedagogically efficacious:

❖ Introduce trainees to qualitative research and help them design qualitative-based individual-based learning.
❖ Help trainees on how to develop appropriate qualitative questions that are not “leading questions” cognizant of the fact that leading questions tend to point interviewees to certain kinds of answers other than those that are authentic and valid in their lives.
❖ Train trainees to have interview skills (how best to ask questions, and acquire listening skills)
❖ Help students identify experts in their area of expertise to enable them interview and learn from them.
❖ See under “*A typical strategy used in a phenomenological study involves”* for data collection and analysis using SBQLA

It is important to emphasize the fact that the methodology presented here is not set in stone. To introduce the SBQLA method to implement any form of innovation in another discipline (be it public health or other), one must customize the methodology to suit the unique needs of the filed. What is most important is for the trainer/instructor to introduce the trainees to the main tenets of qualitative research, especially its methodological approaches in research design, data collection (especially, interviews, narratives, etc.), and find out research participants' lived experiences. The instructor must also guide the students in qualitative research-based analysis (especially, doing content analysis, identifying common themes in participants' responses, and drawing conclusions based on the common themes). The expected trainees' skill-sets identified in the above paragraph are subject to revision/modification to suit the unique needs of specific subject areas and disciplines.

#### Assessment

I begin this section by presenting how the trainees are assessed followed by an assessment of the SBQLA methodology itself.

° In using SBQLA, the assessment include trainees collected excerpts on “lived experiences” sharing them in the form of presentation. Assessment also include writing a paper.
° Presentations and papers are graded based on following rubric.

#### Presentation

➢ The relevance of the topic to the PH field.
➢ Professional introduction of the presenter and her/his topic.
➢ The use of proper headers and captions to arrange information presentation materials
➢ Grasp of content
➢ Students ability to present their field collected information in an appealing manner
➢ Clear oral presentation
➢ Presenter's ability to answer questions.

#### Writing a Paper

Doing a literature about their topic

➢ Providing a detailed description of how the data were collected, coded and analyzed
➢ Describing the findings of the research
➢ Relating the findings to those of the literature; e.g., how similar to or different from your findings are, to those that obtained in the literature (especially, those that you referenced in your literature review); what do these similarities and/or difference suggest now and project into the future research?
➢ What are the limitations of the study?
➢ What is the significance of what learners learned and how the information will be used in their future work

Important questions that help unearth participants' phenomenon include the following:

❖ Can you elucidate your typical day's at work?
❖ What are your memorable experiences about your job?
❖ What are some of the ways that your clients benefitted from these memorable experiences?
❖ What are some of the issues/challenges that you experience that you wish had not happened, and how did you deal with it?
❖ Do you have any experience(s) that you want to share with new workforce that you think will benefit them in their future jobs?
❖ If you happen to get about 10 min to share your experiences with future workforce who will work in your field, what will you tell them?

## Discussion and Conclusions

### Assessment of the SBQLA Methodology Itself: What Makes It an Important Pedagogical Tool?

Research has shown that *didactic approach* to learning is centered on teachers' interest(s) and not information acquired from the field by trainees (2). This teaching method does not make trainees the focus of the teaching/learning process, hence there is the tendency to produce learners whose education is teacher-centered.

However, when we employed SBQLA in our Program, it created creativity among the trainees and made it possible for them to know some of the actual root causes of problems that are causing maternal mortality. The SBQLA pedagogical method also helped PH trainees to apply what they had learned to address real life challenges faced by women.

## Results and Discussion

On the whole, 59 trainees presented papers at national and international conferences and 25 trainees' papers were accepted and published in important refereed journals. It was indeed a great opportunity and experience for the students since, for most of them, that was their first time of writing publishable papers. The “thank you” note cards and letters received from some of the students do not only make the author fulfilled, but also inspire her to provide same assistance and opportunity to other students and to publish the results of this teaching method/idea for others to benefit from.

*Outcomes of the* ***SBQLA*** *training method:*

Students' developed the ability to identify such typical problems as: lack of prenatal care (due to non-existence or close proximity of care facilities), lack of transportation, neglect and bias care experienced by women. Other identified problems included how breastfeeding women experience lack of comfortable places to nurse their children or pump breastmilk for their children thereby leading to mothers using bathrooms as lactation facility. There is no doubt that using bathrooms to pump breastmilk or nurse children is most inappropriate cognizant of the fact that nobody eats their lunch in the bathroom.

### Action Taken by Students to Mitigate the Identified Problems

Problems such as anxiety, depression and breastfeeding among women became the trainees' top priority as issues they plan to work on when employed in the MCH field.

## Outcomes

Sixteen (16) of the trainees decided to “start post-partum groups” in different communities in the US to address some of the neglected needs of new mothers and their families when they enter the workforce.

The SBQLA approach discussed in this paper bolsters Wassinger's (8) *flipped classroom method* and Kowalczyk's (9) *problem-based learning* (PBL) which view trainees' preference for student-centered pedagogy over teacher-centered teaching approach. The findings of this (SBQLA) study, especially, the extent to which the trainees gained professional recognition in being able to present their work at international conferences, is a major achievement. Most importantly, getting their papers accepted and published in reputable journals testifies to the strength of this teaching approach.

Outcome: (a) Another important benefit of the SBQLA approach, as seen from the findings, was trainees' ability to come up with important topics of interest to them; (b) An additional benefit was their ability to develop assessment methods that dealt with the lived experiences of their studied participants during their interviews; (c) Several of the trainees have started initiating research that will help them to understand MCH challenges deeply. Their research addresses such issues as postnatal care, breastfeeding in public, Coronavirus pandemic and MCH issues, teenage pregnancy, substance use in the pandemic and lack of insurance for families. It is anticipated that results from such research will inform their future practice.

SBQLA take us into the “minds” and lived experiences of the studied participants to unearth their innermost thoughts, feelings, fears, joy, among others. This makes the methodology an important enterprise in social science. The objectivity of the method must be judged in the extent to which it places the researcher in particular, and public health practitioners in general, at the same camera angle of the studied “communities” to experience their unique phenomena and to understand what is going on in order to help come up with workable and acceptable and culturally congruent solutions.

### Exit Interviews

Based on exit interviews over 90% of the students noted that their research experience *via* SBQLA was a great opportunity and an experience for them. Twenty-one (21) students noted that it was their first time of writing a publishable paper. Ten students sent “thank you” note cards and letters to show appreciation for what they learned using SBQLA. What is important is the fact that this shows the approach works and that if carefully customized it can be applied to various disciplines or sub-disciplines.

### Limitations

Although this approach of teaching is beneficial, it is also time consuming in the sense that the teacher and trainees had to make several contacts with experts in the field. This was initially challenging for some of the students, particularly, due to schedule conflicts. Note, however, that no matter the difficulty involved in identifying and contacting the studied participants, the SBQLA approach provided the trainees with a great academic opportunity and professional experience. For some, it was their first time of making academic and/or professional contact for learning from experts in the field.

## Conclusion and Implications of the Study

In conclusion, the SBQLA made it possible for MCH experts to shed light on true stories shared by real and authentic individuals whose faces could be associated with their shared experiences. Employing SBQLA has made it possible for students to present some of their work at national conferences. They have produced data that will help guide future MCH workforce trainees. It has also made it possible for trainees to initiate several projects that will help them tackle existing and emerging issues in MCH in their future work.

This study has implications for pedagogy in general, and for public health pedagogy in particular. In particular, it urges public health pedagogues to look beyond traditional teaching methodologies and to come up with novel and innovative methods that put trainees at the center of the training process. By so doing, public health trainees will become more aware of their MCH professional environments, ask the important health questions of *what, why*, and *how* and come up with solutions that interrogate and are interrogated by the needs of parents and their children/infants. Finally, this SBQLA methodology has implications for the professional advancement of public health trainees in the area of research publication. The fact that trainees are trained in academic writing puts them in a position where they can competently and effectively apply and compete for academic and professional positions at the end of their training.

## Data Availability Statement

The original contributions presented in the study are included in the article, further inquiries can be directed to the corresponding author.

## Ethics Statement

Ethics approval Protocol Number: 1403865787.

## Author Contributions

CO conceived the idea and wrote the manuscript.

## Conflict of Interest

The author declares that the research was conducted in the absence of any commercial or financial relationships that could be construed as a potential conflict of interest.

## Publisher's Note

All claims expressed in this article are solely those of the authors and do not necessarily represent those of their affiliated organizations, or those of the publisher, the editors and the reviewers. Any product that may be evaluated in this article, or claim that may be made by its manufacturer, is not guaranteed or endorsed by the publisher.
